# The regulatory effect of growth differentiation factor 11 on different cells

**DOI:** 10.3389/fimmu.2023.1323670

**Published:** 2023-12-08

**Authors:** Yingchun Shao, Ting Liu, Xiaobo Wen, Renshuai Zhang, Xinlin Liu, Dongming Xing

**Affiliations:** ^1^The Affiliated Hospital of Qingdao University, Qingdao University, Qingdao, China; ^2^Qingdao Cancer Institute, Qingdao, China; ^3^School of Life Sciences, Tsinghua University, Beijing, China

**Keywords:** growth differentiation factor 11, GDF11, cells, diseases, function

## Abstract

Growth differentiation factor 11 (GDF11) is one of the important factors in the pathophysiological process of animals. It is widely expressed in many tissues and organs of animals, showing its wide biological activity and potential application value. Previous research has demonstrated that GDF11 has a therapeutic effect on various diseases, such as anti-myocardial aging and anti-tumor. This has not only sparked intense interest and enthusiasm among academics but also spurred some for-profit businesses to attempt to develop GDF11 as a medication for regenerative medicine or anti-aging application. Currently, Sotatercept, a GDF11 antibody drug, is in the marketing application stage, and HS-235 and rGDF11 are in the preclinical research stage. Therefore, we believe that figuring out which cells GDF11 acts on and its current problems should be an important issue in the scientific and commercial communities. Only through extensive, comprehensive research and discussion can we better understand the role and potential of GDF11, while avoiding unnecessary risks and misinformation. In this review, we aimed to summarize the role of GDF11 in different cells and its current controversies and challenges, providing an important reference for us to deeply understand the function of GDF11 and formulate more effective treatment strategies in the future.

## 1 Introduction

Growth differentiation factor 11 (GDF11) is a secreted protein cloned from dental pulp. In 1956, Clive McCay fused the blood circulation systems of young mice with those of old mice by establishing a “parabiosis” system. The results showed that the old mice began to “reverse growth”, whereas the young mice began to age before they were old. The news immediately caused an uproar in the scientific community; however, at this time the underlying mechanism of the phenomenon was not elucidated. In 2013, Wagers team offered a possible explanation for this “reverse growth” phenomenon. They reported that the systemic levels of GDF11 decreased significantly in older mice compared with those in younger mice. When GDF11 was injected into old mice, heart failure, cerebrovascular depletion, and skeletal muscle dysfunction due to aging were significantly improved (1–3). Since then, GDF11 has become a known growth factor. However, these results were later challenged by studies showing that blood GDF11 levels in aging animals were on the rise. Restoring GDF11 levels exhibited no significant effect on cardiac functions and structures, and skeletal muscle function in aging animals was impaired (4–6). Subsequently, both positive and negative effects of GDF11 were reported one after another.

GDF11 was first reported in 1999 and is considered to be a crucial signaling molecule in embryonic development (7). Since then, its different functions have been gradually discovered. Studies have shown that GDF11 is associated with physiological and pathological processes, such as tumor growth (8–10), organ development (11–13), aging (14–16), and nervous system (17–19). GDF11 has powerful physiological functions. However, with the advancement of research, the controversial information on GDF11 has increased. Therefore, to better investigate the role of GDF11 and increase its applicability, we summarized the role of GDF11 in different cells and the development of drugs targeting GDF11, hoping to provide an important reference for us to deeply understand the function of GDF11 and formulate more effective treatment strategies in the future.

## 2 Discovery and formation of GDF11

**Figure 1:**
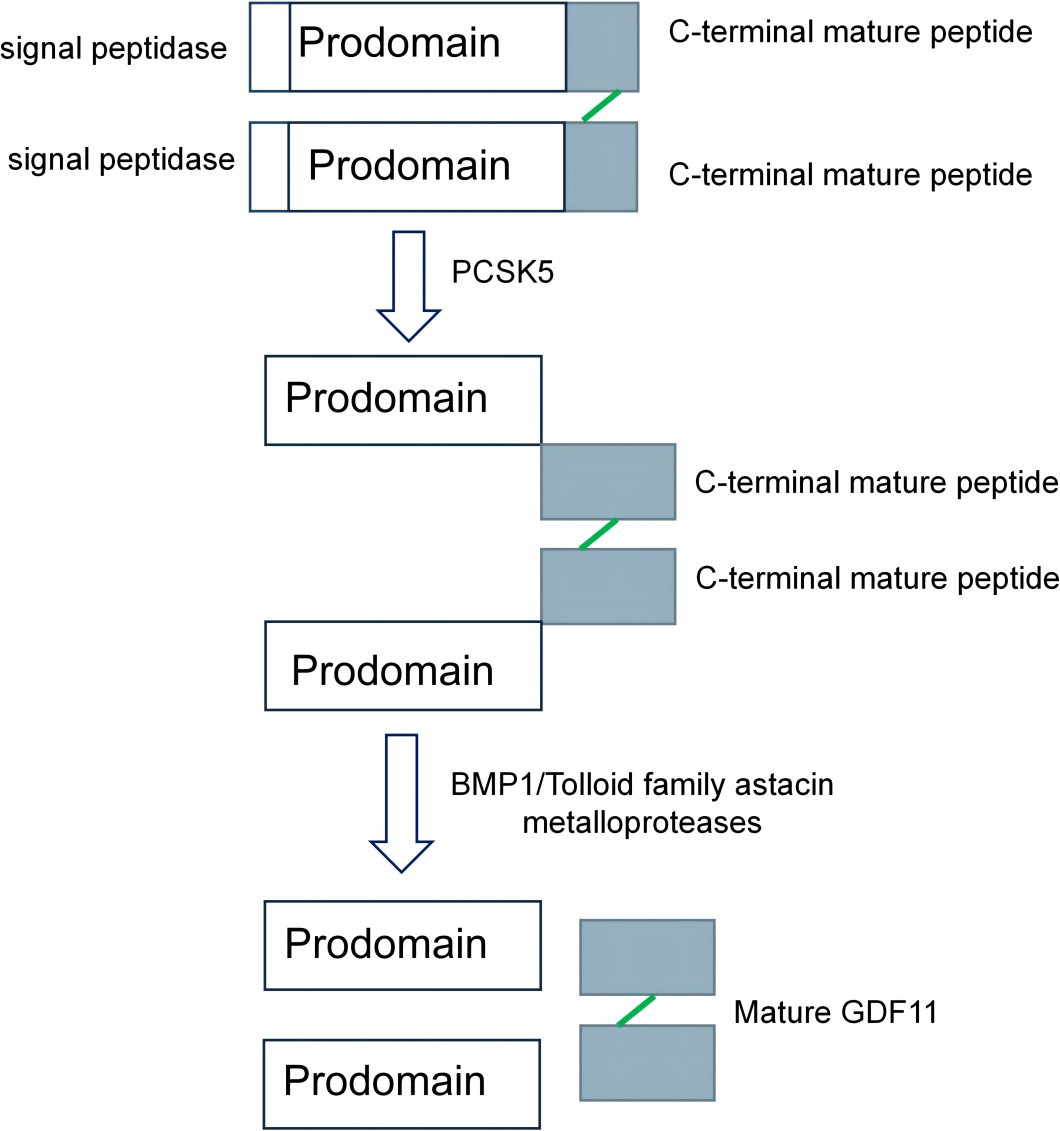
The formation process of growth differentiation factor 11 (GDF11). The precursor protein of GDF11 is hydrolyzed by proprotein convertase subtilisin/kexin 5/(PCSK5), which removes the preregion sequence of the precursor protein and forms a non-covalently linked latent complex. It is then sheared by BMP1/Tolloid family astacin metalloproteases to form mature GDF11 (21, 22).

GDF11 was first amplified from the pulp of a mouse incisor using a reverse transcriptase chain reaction by Nakashima et al. (7). The peptide segment in the mature region shared 90% homology with growth differentiation factor 8 (20). The formation of GDF11 is mainly divided into the following two steps: First, the mRNA of GDF11 is initially translated to form a precursor protein, which is hydrolyzed by the proteolytic enzyme proprotein convertase subtilisin/kexin 5 (PCSK5) to form a non-covalently bonded latent complex. The complex is then activated by the metal-proteinases of the development-related BMP1/Tolloid family to form mature GDF11 after being cleaved at specific sites (**Figure 1**) (21, 22).

## 3 The role and controversy of GDF11 in different cells

### 3.1 Cancer cells

**Figure 2:**
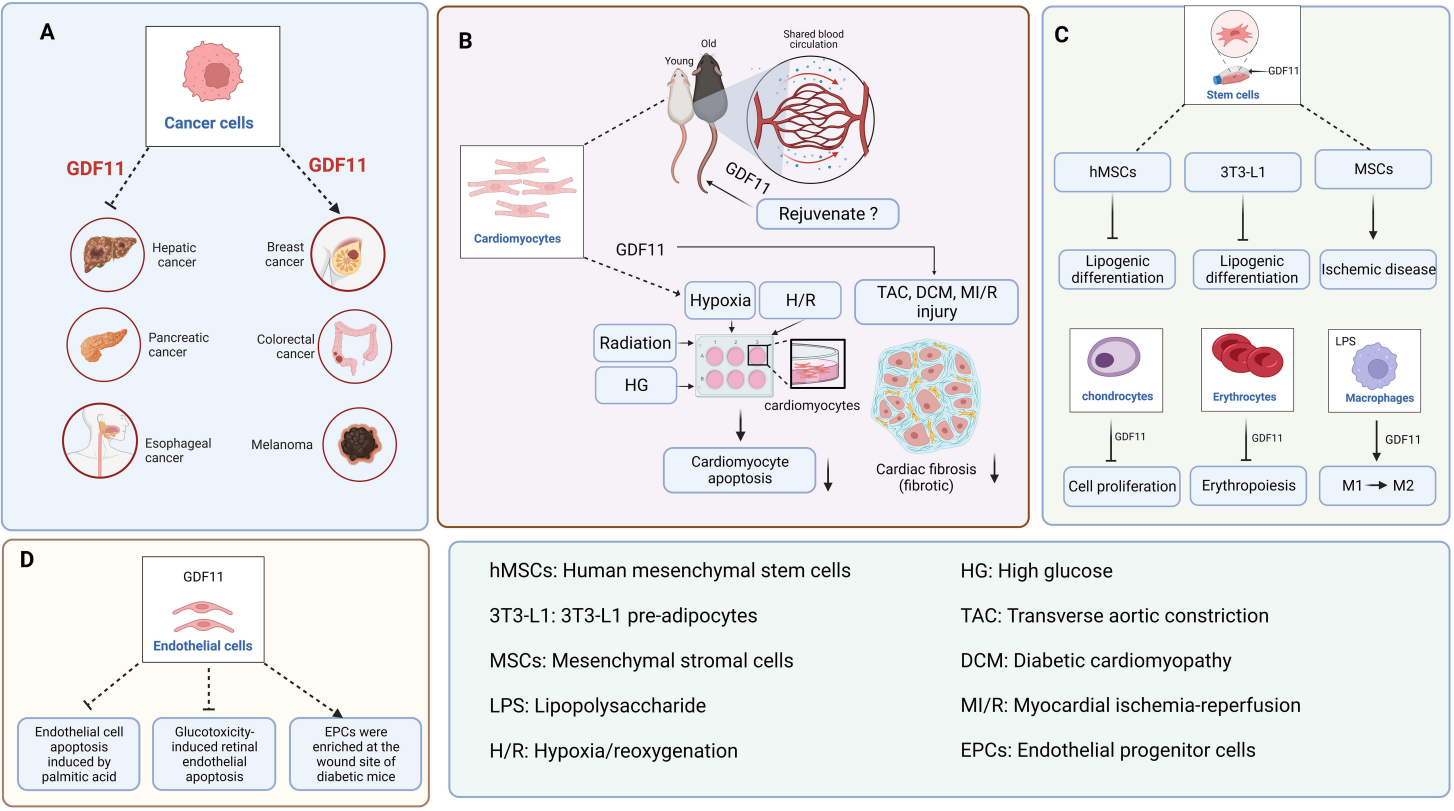
The function of GDF11 in various cells. **(A)**. GDF11 is involved in the occurrence and development of liver cancer, pancreatic cancer, esophageal cancer, breast cancer, colon cancer, and melanoma. **(B)**. The regulatory effect of GDF11 on cardiomyocytes. **(C)**. The regulatory effects of GDF11 on stem cells, chondrocytes, erythrocytes, and macrophages. **(D)**. The regulatory effect of GDF11 on endothelial cells. Created with BioRender.com.

GDF11 can inhibit the growth of some tumors; however, it has the opposite role in other tumors (**Figure 2A**). For instance, GDF11 levels are increased in colon cancer (23), breast cancer (24), oral squamous cell carcinoma (25), and melanoma (26, 27). The upregulation of GDF11 can promote the occurrence and development of related tumors. In liver cancer (28, 29), pancreatic cancer (8), esophageal cancer (30), and cholangiocarcinoma (31), the expression of GDF11 is downregulated. However, when GDF11 is overexpressed, the progression of the above tumors is inhibited. Furthermore, lung cancer is the most common cancer, and only two studies have shown whether GDF11 exerts a therapeutic effect on it. Among them, Marini et al. showed that GDF11 plays an important role in the development of congenital platinum resistance in lung adenocarcinoma. They reported that inhibiting GDF11 expression can effectively overcome the development of intrinsic platinum resistance in lung adenocarcinoma (32). Contrastingly, a study by Lim et al. reported that GDF11 is not a reliable biomarker for predicting the effectiveness of platinum-based chemotherapy for advanced non-small cell lung cancer because no significant correlation was found between its overexpression and the overall survival of patients (33). Therefore, it can be concluded that the role of GDF11 in different types of cancer is different.

### 3.2 Cardiomyocytes

Recently, the most controversial issue of GDF11 has been its age-related expression and anti-aging role. Wagers team reported that GDF11 expression was downregulated with age, and exogenous administration of GDF11 can reverse age-related cardiac hypertrophy (1). Subsequently, the team reported that GDF11 can repair damaged skeletal muscle and reverse age-related cognitive dysfunction (2, 3). However, based on western blotting, RNA expression, and GDF11-specific immunoassay, Egerman et al. reported that GDF11 expression in the blood may actually increase with age (6). Meanwhile, when they regularly injected GDF11 into mice with bone injuries, and found that GDF11 did not induce muscle regeneration (34). Similarly, Hinken et al. reached the same conclusion as that by Egerman et al. and they unanimously overruled the argument by the Wagers team that GDF11 can reverse age-related skeletal muscle and stem cells dysfunction (35). These findings contradict the report by the Wagers team that the expression of GDF11 in the blood decreases with age and promotes muscle stem cells regeneration. Furthermore, Smith et al. reported that GDF11 did not save the pathologic myocardial hypertrophy associated with aging but accelerated the aging process (4). Harper et al. reported that GDF11 decreases pathological hypertrophy during stress overload (36). These findings are contrary to those of the Wagers team. In response, the Wagers team consistently explained that Egerman et al. mistakenly identified the immunoglobulin bands as GDF11 bands, and the immunoglobulin increases with age (37). Moreover, the inconsistency with the results obtained by Smith et al. may be due to the large batch variation in the concentration of recombinant GDF11 protein. Significantly, GDF11 can alleviate cardiomyocyte damage caused by pathological conditions such as hypoxia (38, 39), hypoxia/reoxygenation (40–42), high glucose (43, 44), and radiation (45) (**Figure 2B**).

### 3.3 Fibroblasts

Several studies have shown that GDF11 is associated with the specific fibrosis of the heart, kidney, liver, skin, and other organs. GDF11 exhibits an anti-myocardial fibrosis effect in transverse aortic constriction (TAC) surgery (36), diabetic cardiomyopathy (DCM) (44), and myocardial ischemia-reperfusion (MI/R) injury (**Figure 2B**) (41). However, the role of GDF11 in liver fibrosis is controversial. Dai et al. reported that GDF11 exhibited an inhibitory effect on liver fibrosis, while Frohlich et al. found that GDF11 could induce mild hepatic fibrosis (46, 47). We believe that the opposite conclusions drawn by the two articles may be due to the differences between the two models. Dai et al. discussed metabolism-related liver fibrosis, while Frohlich et al. simply used CCl_4_ to induce mouse liver fibrosis model, so the two models are essentially different. Additionally, GDF11 exhibits a pro-fibrotic effect on kidney fibroblasts (48, 49) and skin fibroblasts (50, 51). Therefore, the role of GDF11 depends on the studied organs and diseases and should be studied and analyzed in detail in a specific setting.

### 3.4 Macrophages

The significance of GDF11 in the immune system, particularly in macrophages, has been discovered in recent years (**Figure 2C**). Research studies have shown that GDF11 can facilitate the conversion of M1 macrophages into M2, which can ultimately decrease inflammatory responses (52, 53). Furthermore, GDF11 can promote the metabolic regulatory function of macrophages. For instance, GDF11 is capable of facilitating the removal of cholesterol from macrophages by suppressing peroxisome proliferator-activated receptor (PPAR)-γ expression (54).

### 3.5 Stem cells

Stem cells therapy has broad application prospects for numerous diseases. Previous studies have shown that GDF11 can improve the effectiveness of stem cells transplantation (55, 56). For instance, GDF11 plays a therapeutic role in cardiovascular system diseases and nervous system diseases via the regulation of MSCs and neural stem cells (NSCs). MSCs can be used for treating ischemic diseases to promote angiogenesis has been extensively carried out (**Figure 2C**) (57–59). However, the regulatory function of GDF11 on NSCs are controversial. Katsimpardi et al. reported that circulating GDF11 in the blood can increase the proliferation and differentiation of NSCs (60). However, Wang and Williams et al. reported that GDF11 can inhibit the proliferation and migration of NSCs while facilitating apoptosis and differentiation (61, 62). Owing to the contradiction between these two views, Wang et al. reported that the effect of circulating GDF11 on NSCs was indirect when they added GDF11 directly to NSCs. Thus, GDF11 appears to be a “pro-aging factor” when it acts directly on NSCs, whereas a “rejuvenating factor” when it acts indirectly on NSCs.

### 3.6 Red blood cells

GDF11 is a regulator of erythropoiesis (**Figure 2C**). Previous studies have shown that GDF11 can lead to mild anemia (63), and downregulating GDF11 expression can help treat thalassemia (64). Furthermore, the overexpression of GDF11 in the blood of patients with myelodysplastic syndrome can inhibit the production of red blood cells and exacerbate the condition of the patients (65).

### 3.7 Bone-related cells

GDF11 could repair age-induced skeletal muscle dysfunction (2). However, the function of GDF11 was questioned soon, and several studies confirmed that GDF11 could not restore the function of damaged skeletal muscle in aged mice but significantly inhibited the regeneration of skeletal muscle cells in mice (66–68). Along with its effects on skeletal muscle cells, GDF11 is also considered to exhibit a certain regulatory effect on the growth and differentiation of osteoblasts, osteoclasts, and chondrocytes. In 2015, Zhang et al. reported that GDF11 is a promoter of osteoblasts formation (69). However, Lu, Liu, and Shen et al. reported that GDF11 can inhibit the differentiation of bone marrow mesenchymal stem cells (BMSCs) into osteoblasts (70–72). Although Lu and Liu et al. agreed that GDF11 can inhibit the differentiation of BMSCs into osteoblasts, they reported inconsistent conclusions on the regulation of osteoclasts. The experimental results by Lu et al. showed that GDF11 did not have a significant effect on osteoclasts differentiation, whereas Liu et al. showed that GDF11 could promote osteoclasts formation. Furthermore, a recent study reported that GDF11 can exacerbate hip dysplasia via the inhibition of chondrocytes proliferation and hypertrophy (**Figure 2C**) (73). Thus, it can be concluded that the regulatory role of GDF11 on osteoblasts and osteoclasts is intricate.

### 3.8 Adipocytes

Previous studies have shown that GDF11 may be a core factor regulating the balance of osteoblasts–adipocytes differentiation. However, in elderly patients with osteoporosis, whether GDF11 promotes or inhibits the differentiation of BMSCs into adipocytes is not known. Shen et al. reported that GDF11 expression in the serum of elderly patients with osteoporosis was upregulated, which promoted the adipogenic differentiation of BMSCs (71). However, Zhang et al. reported that the expression of GDF11 in the serum of elderly patients with osteoporosis was decreased, which could inhibit the adipogenic differentiation of BMSCs (69). Similarly, Luo et al. reported that GDF11 inhibited the adipogenic differentiation of human mesenchymal stem cells (hMSCs) and 3T3-L1 pre-adipocytes via the activation of Smad2/3 pathway (**Figure 2C**) (74). Frohlich et al. also confirmed that GDF11 inhibited the adipogenic differentiation of 3T3-L1 pre-adipocytes (75). Furthermore, GDF11 also exerts a regulatory role in the function of human adipose-derived stromal cells (HADSCs). GDF11 inhibits the adipogenic differentiation of HADSCs (76). GDF11 decreases the content of triglycerides in adipocytes by inhibiting adipocytes anabolism and promoting fat catabolism (77). To summarize, although the role of GDF11 in the differentiation of BMSCs into adipocytes in elderly patients with osteoporosis is controversial, the abovementioned findings indicate that GDF11 may be a potential target for inhibiting adipogenic differentiation and treating obesity.

### 3.9 Endothelial cells

GDF11 can alleviate the pathological progression of atherosclerosis, diabetic wounds, and diabetic retinopathy by improving endothelial cells dysfunction (**Figure 2D**). For instance, GDF11 can decrease the area of atherosclerotic plaques by decreasing palmitic acid-induced apoptosis of endothelial cells (54). GDF11 can promote the enrichment of endothelial progenitor cells (EPCs) in the wounds of diabetic mice, thus accelerating wound healing (78, 79). GDF11 can alleviate the dysfunction of glucotoxicity-induced retinal microvascular endothelial cells in mice, thereby decreasing the progression of diabetic retinopathy (80). Furthermore, the effect of GDF11 on endothelial cells treated with/without serum was different. Zhang et al. reported that GDF11 exhibited no significant effect on the proliferation, migration, and death of human umbilical vein endothelial cells (HUVECs) under serum-rich conditions but increased the viability of HUVECs under serum-free conditions (81). The purpose of using serum-free is to remove the interference of cytokines in serum. Finkenzeller et al. examined the effect of GDF11 on the migration and tube formation of peripheral blood endothelial progenitor cells under serum-free conditions. The experimental results indicated that GDF11 has a promoting effect on this (82). Thus, GDF11 can improve the dysfunction of HUVECs; however, the degree of influence on HUVECs was different in culture conditions with/without serum.

To summarize, GDF11 is an important growth factor that can be regulated in different types of cells (**Figure 2**). Therefore, GDF11 has garnered increasing attention in many research fields, and its research is expected to help us better understand and treat several diseases.

## 4 GDF11-based therapeutic strategies

GDF11 is a crucial growth factor and exerts therapeutic effects on a variety of diseases (**Table 1**). Through our comprehensive summary of GDF11, we know that current studies on the treatment of GDF11 mainly focus on the following aspects:1) increase the expression of GDF11 or adjuvant therapy of GDF11. This may include increasing GDF11 expression through gene therapy or pharmacological intervention. The goal of these approaches is to treat specific diseases by increasing the activity of GDF11 to promote tissue repair and regeneration. 2) GDF11 was used as a marker to evaluate the risk and prognosis of diseases. By detecting the level of GDF11, the severity of diseases can be assessed and the progression of diseases can be predicted, which provides a theoretical basis for individualized treatment plans. For example, for some diseases, high levels of GDF11 may indicate a more severe condition, while low levels of GDF11 may be associated with a better prognosis. 3) Research and development of new drugs with GDF11 as the target. As for the research on drugs targeting GDF11, there are currently some drugs under development, such as rGDF11, sotatercept, and HS-235, which are all new drugs developed targeting GDF11 (**Table 2**). These drugs affect related cellular and physiological processes mainly by regulating GDF11 expression. In summary, research on GDF11 treatment mainly focuses on changing the expression of GDF11, evaluating its value as a marker, and developing new drugs targeting GDF11. These studies are of great significance for revealing the mechanism of action of GDF11 in disease treatment and developing new treatment strategies.

**Table 1:** Positive effects of GDF11.

Cell type	Disease model	GDF11 expression	Main function	References
**Cancer cells**	Liver cancer	↓	GDF11 can inhibit liver cancer progression	(28, 29)
	Pancreatic cancer	↓	GDF11 can inhibit pancreatic cancer progression	(8)
	Esophageal cancer	↓	GDF11 can inhibit esophageal cancer progression	(30)
	Cholangiocarcinoma	↓	GDF11 can inhibit cholangiocarcinoma progression	(31)
**Cardiomyocytes** **Cardiomyocytes**	Aging mouse	↓	GDF11 can reverse age-related cardiac hypertrophy	(1)
	Myocardial infarction (MI)	↓	GDF11 can improve heart function in MI mice	(38, 39)
	Diabetic cardiomyopathy (DCM)	↓	GDF11 can alleviate pathological myocardial remodeling in DCM	(44)
	Myocardial ischaemia/reperfusion (MI/R) injury	↓	GDF11 can attenuate MI/R injury	(40–42)
	Mice received Gray whole-heart irradiation	↓	GDF11 can significantly mitigate cardiac radiotoxicity	(42)
**Fibroblasts**	Transverse aortic constriction (TAC)	↓	GDF11 can inhibit myocardial fibrosis induced by TAC	(36)
	DCM	↓	GDF11 can inhibit myocardial fibrosis induced by DCM	(44)
	MI/R injury	↓	GDF11 can inhibit myocardial fibrosis induced by MI/R injury	(41)
**Macrophages** **Macrophages**	Severe acute pancreatitis	↑	GDF11 facilitates the conversion of M1 macrophages into M2, thereby improving severe acute pancreatitis	(52)
	Atherosclerosis	−	GDF11 can reduce inflammatory cytokines expression in macrophages	(54)
	Acute kidney injury (AKI)	↑	GDF11 alleviates AKI injury through regulating the polarization of M1/M2 macrophages	(53)
**Mesenchymal stem cells (MSCs)**	Hypoxic-induced MSCs	↓	GDF11 protects cardiac MSCs from apoptosis under hypoxic condition	(55)
**Satellite cells**	Age-related dysfunction in mouse skeletal muscle	−	GDF11 can improve muscle physiology and physical function in aged mice	(2)
**Osteoblasts**	Osteoporosis	↓	GDF11 can induce osteoblastogenesis and related gene expression	(69)
**3T3-L1 white and HIB1B brown adipocytes**	Obesity	↑	GDF11 can enhance the thermogenesis of white adipocytes and weaken adipogenesis	(77)
**Endothelial cells**	Atherosclerosis	−	GDF11 decreases the area of atherosclerotic plaques by decreasing palmitic acid-induced apoptosis of endothelial cells	(54)
	Non-healing diabetic wounds	↓	GDF11 promotes the enrichment of endothelial progenitor cells in the wounds of diabetic mice, thus accelerating wound healing	(78, 79)
	Diabetic retinopathy disease	−	GDF11 alleviates the dysfunction of glucotoxicity-induced retinal microvascular endothelial cells in mice	(80)

MSCs, Mesenchymal stem cells; MI, Myocardial infarction; DCM, Diabetic cardiomyopathy; MI/R, Myocardial ischaemia/reperfusion; AKI, Acute kidney injury. ↑: GDF11 expression increased; ↓: GDF11 expression decreased; -: No detection were added.

**Table 2:** Drug development targeting GDF11.

Drug names	First R&D enterprise	Targets	Indications	Development Phase	Drug types
Sotatercept	Acceleron Pharma Inc	ACVR2 ACVR2A GDF11	Beta thalassaemia Myelofibrosis Pulmonary arterial hypertension	Apply for going public	Fragment antibody Immunoglobulin G homologous antibody Chimeric protein Soluble receptor
HS-235	35Pharma Inc	ACVR2A GDF11 GDF8	Obesity	Preclinical	Fusion protein
rGDF11	Harvard University	GDF11	Alzheimer disease Diseases of coronary artery Type 2 diabetes mellitus Cerebral ischaemic stroke	Preclinical	Recombinant protein

Information source: https://pharma.bcpmdata.com/.

## 5 Conclusions and prospects

**Figure 3:**
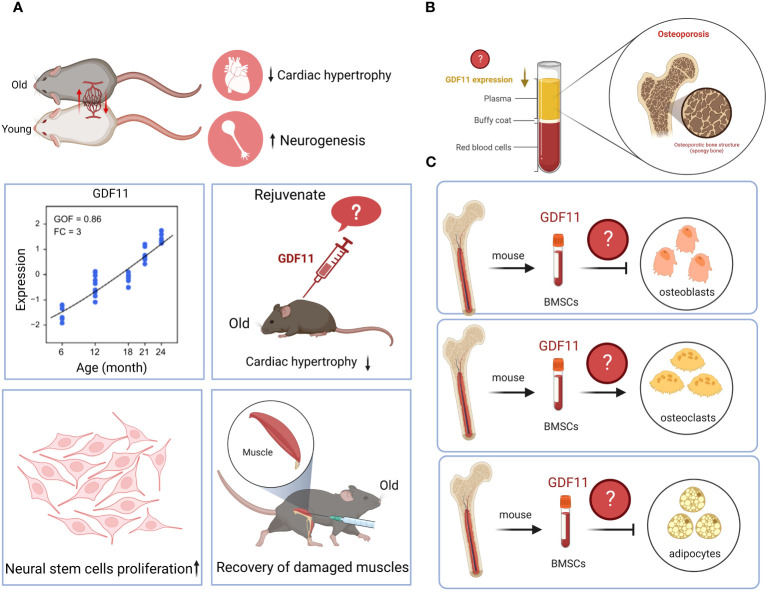
Controversy surrounding GDF11. **(A)**. Whether GDF11 has anti-aging effects? **(B)**. Is the serum expression of GDF11 low or high in elderly patients with osteoporosis? **(C)**. What role does GDF11 play in the differentiation of bone marrow mesenchymal stem cells into osteoblasts, osteoclasts, and adipocytes? Created with BioRender.com.

GDF11 has a wide range of biological activities and has therapeutic effect on many diseases. It can inhibit the progression of liver cancer, pancreatic cancer, esophageal cancer, and cholangiocarcinoma; can protect against myocardial damage caused by MI, MI/R, DCM, TAC and radiation; can reduce inflammatory responses and improve endothelial cells dysfunction. However, there is currently some controversy surrounding GDF11, which hinders the research and application of GDF11. Therefore, a broad and comprehensive discussion will contribute to a better understanding of the role and potential of GDF11. In this review, we provide an in-depth exploration of the GDF11 function and its controversy, which may inform future studies on the biological role of GDF11 and its potential limitations in disease treatment and prevention. Based on the abovementioned summary, the current controversy over GDF11 is as follows (**Figure 3**): 1. Whether GDF11 has anti-aging effects? 2. Is the serum expression of GDF11 low or high in elderly patients with osteoporosis? 3. What role does GDF11 play in the differentiation of bone marrow mesenchymal stem cells into osteoblasts, osteoclasts, and adipocytes? We believe that the reason for the controversy may be due to the complex biological regulation mechanism of GDF11 and the different responses of different types of cells and tissues to GDF11. The dose, route, and interval of administration used by different research teams are inconsistent, which also causes different effects of GDF11 *in vivo*. The signaling pathway mediated by GDF11 is complex and changeable, and its complex cross-action *in vivo* can lead to inconsistency in results. Additionally, the inconsistent results may be related to factors such as sample selection, research methods, and experimental techniques. To resolve these controversies, we suggest that future studies should adopt more accurate and consistent experimental methods to obtain more reliable conclusions. Meanwhile, we believe that researchers need to further explore the optimal therapeutic dose, route and time window of GDF11 to maximize its therapeutic potential and gain insight into the mechanism of action of GDF11, which can help us determine how GDF11 acts in different tissues and disease states, and provide a basis for optimizing treatment strategies. In addition, researchers can also try to develop GDF11 activators with better selectivity to reduce the impact on other related molecules, thereby improving the specificity and safety of the treatment. Although the above points of GDF11 are still uncertain, it is undeniable that the emergence of GDF11 brings unlimited hope to humans for delaying aging, resisting diseases and even tumors.

In general, GDF11 is a powerful biomolecule that has immense research significance. Just because of different research methods, GDF11 often shows an elusive double-edged sword effect, which also puts a mysterious veil on its research. Therefore, further studies are required in the future to verify and optimize its therapeutic effect and safety and to overcome its potential double-edged sword effect to achieve its complete clinical application. But we firmly believe that with the continuous updating of current experimental technology, the secrets hidden by GDF11 will gradually be revealed, and its powerful functions will ultimately bring benefits to mankind.

## Author contributions

YS: Conceptualization, Writing – original draft, Investigation. TL: Investigation, Writing – review & editing. XW: Writing – review & editing, Investigation. RZ: Writing – review & editing, Conceptualization. XL: Conceptualization, Writing – review & editing. DX: Validation, Writing – review & editing.
